# Atlanta Polyclinic

**Published:** 1893-04

**Authors:** 


### ATLANTA POLYCLINIC.

The formal opening of the Atlanta Polyclinic occurred on Wednesday March 22d at their building on the corner of Marietta and Magnolia streets and was well attended by the profession and other friends of the institution.

After prayer by Rev. Dr. Kendall, the President, Dr. F. W. McRae in a brief address outlined the aims and objects of the Polyclinic and dispelled some of the erroneous ideas existing concerning the college, showing that its existence aided other charitable institutions, harmonizing and advancing their interests as well as the other medical schools of Atlanta and the South, and that the large number of patients treated daily at the Polyclinic demonstrated that Atlanta with its railroad facilities, factories, etc., would furnish abundant clinical material for such a school.

At the conclusion of his remarks Hon. J. B. Goodwin, Mayor of Atlanta, and Dr. J. D. Turner, President of the Atlanta Charitable Association, made brief addresses, giving assurance of good will and promising hearty co-operation in the endeavor to elevate the standard of medical education.

These distinguished gentlemen were followed by the address of the occasion delivered by Mr. F. H. Richardson, Editor of *The Evening Journal*, who upheld all tendencies toward mental advancement with special reference to medicine, he welcomed the establishment of such institutions believing them to be productive of more good than factories or any mechanical measures to this city and the South.

The building was then thrown open for the inspection of visitors. A more favorably situated and well arranged building would have been very difficult to find. The first floor is utilized for practical work where seventy-five to one hundred and twenty-five patients are now treated daily on this floor ; we find a large well-lighted operating room, lecture room, patients waiting room, dispensary, reading room, dark room, and laboratory.

The second floor is divided into wards for patients requiring surgical operations. Quite a number of interesting operations have already been performed, among them we may mention Cœliotomy for Extra-uterine Pregnancy, removal of Sup. Maxilla, extirpation of Scirrhus Breast, Hemorrhoids, diseased Lymphatics, Circumcisions &c., &c.

The Polyclinic will be open all the year, and physicians attending now will have exceptional advantages while the classes are small and the clinics large they can see what is being done and often aid in operations. Students will be shown through the wards daily to see the after treatment of patients operated upon and watch the results being often more important than the operation.

To the busy practitioner and the recent graduate the Polyclinic offers excellent opportunities for reasonable rates at which they can matriculate any time during the year.

